# Familial Chylomicronemia Syndrome Due to a Homozygous LPL c.644G>A Variant and Response to Volanesorsen in Colombia: A Case Report

**DOI:** 10.7759/cureus.111306

**Published:** 2026-06-22

**Authors:** Carlos Berrocal, Juliana Charria Rodríguez, Antonio M Zumaque Carrascal, Danna Vanessa Jimenez Gomez, Elvis Mauricio Pérez Lafont, Mauricio Andres Robayo Perez, Andres Felipe Garcia-Ramos

**Affiliations:** 1 Internal Medicine, Universidad del Valle, Cali, COL; 2 Faculty of Medicine, Universidad del Valle, Cali, COL; 3 General Medicine, Universidad del Sinú, Montería, COL; 4 General Medicine, Empresa Social del Estado (ESE) Vida Sinú, Montería, COL; 5 General Medicine, Clínica IMAT Oncomédica Auna, Montería, COL; 6 Endocrinology, Universidad del Valle, Cali, COL

**Keywords:** acute pancreatitis, diagnostic genetic testing, familial chylomicronemia syndrome, hyperlipoproteinemia type i, hypertriglyceridemia (htg), lipoprotein lipase, precision-medicine

## Abstract

Familial chylomicronemia syndrome (FCS) is a rare genetic disorder characterized by persistent severe hypertriglyceridemia and recurrent acute pancreatitis, and is frequently misdiagnosed as multifactorial hypertriglyceridemia.

We report the case of a 57-year-old Colombian male with recurrent acute pancreatitis and severe hypertriglyceridemia peaking at 4,350 mg/dL, refractory to fibrates, statins, and omega-3 fatty acids, with persistently low low-density lipoprotein (LDL) and high-density lipoprotein (HDL) levels. Secondary causes were excluded, and chronic valproic acid therapy was identified as a potential aggravating factor. A Moulin score of 10 points classified FCS as highly probable, and next-generation sequencing confirmed a homozygous pathogenic *LPL* variant, NM_000237.3.644G>A, p.(Gly215Glu), associated with loss of enzymatic activity. Following diagnostic confirmation, volanesorsen produced a sustained triglyceride reduction, with no new episodes of pancreatitis during uninterrupted therapy.

This case underscores the importance of suspecting FCS in patients with refractory severe hypertriglyceridemia and recurrent pancreatitis. Molecular diagnosis enables access to precision medicine-based strategies with a meaningful impact on clinical outcomes and quality of life.

## Introduction

Familial chylomicronemia syndrome (FCS) is an ultra-rare autosomal recessive disorder caused by biallelic pathogenic variants in genes involved in the lipoprotein lipase (LPL) pathway, including *LPL*, *APOC2*, *APOA5*, *LMF1*, and *GPIHBP1*, resulting in near-complete loss of LPL activity, persistent severe hypertriglyceridemia, and recurrent acute pancreatitis [1,2]. Despite its distinct pathophysiology, FCS is frequently misclassified as multifactorial hypertriglyceridemia, leading to prolonged diagnostic delays and ineffective conventional therapy. We report the case of a 57-year-old Colombian male with refractory hypertriglyceridemia and recurrent pancreatitis in whom next-generation sequencing identified a homozygous pathogenic *LPL* variant, NM_000237.3.644G>A, p.(Gly215Glu), also known by its legacy designation p.Gly188Glu, confirming FCS, with a subsequent marked and sustained biochemical response to volanesorsen, underscoring the value of molecular diagnosis and precision medicine in this condition.

## Case presentation

We report the case of a 57-year-old male, a former farmer from the municipality of Mercaderes, Cauca, Colombia. His surgical history was notable for cholecystectomy performed in 2017. His medical history included a psychiatric comorbidity managed with valproic acid, sertraline, and levomepromazine. Family history was significant for the early deaths of two siblings at ages 17 and 22 years from unclear causes. At the time of presentation, the patient was on chronic rosuvastatin 40 mg nightly and long-term valproic acid therapy.

On November 28, 2019, he presented to the ED with a three-week history of epigastric pain radiating to the right upper quadrant, progressively worsening in intensity. In the hours before admission, the pain became more severe and was accompanied by multiple episodes of vomiting and unquantified fever, prompting medical evaluation.

On admission, the patient was hemodynamically stable, conscious, and oriented. Physical examination revealed deep epigastric tenderness without signs of peritoneal irritation, palpable masses, or visceromegaly; the remainder of the examination was unremarkable.

Initial laboratory tests showed elevated serum lipase (243 U/L) and amylase (141 U/L), along with markedly elevated CRP (149.3 mg/L), suggesting an acute inflammatory process. During sample processing, the laboratory noted a markedly lipemic and lactescent plasma appearance, prompting repeat blood sampling. The second analysis documented a triglyceride level of 1,294 mg/dL, a key finding that guided the etiological workup.

Abdominal ultrasound revealed hepatic steatosis and a nodular lesion in the pancreatic tail. Magnetic resonance cholangiopancreatography subsequently confirmed findings consistent with acute pancreatitis involving the pancreatic head, classified as Balthazar grade C, without evidence of necrosis or fluid collections. Biliary etiology was excluded given the prior cholecystectomy and the absence of choledocholithiasis or biliary duct dilation.

The episode was classified as mild acute pancreatitis and managed conservatively with IV hydration and analgesia, resulting in clinical improvement and discharge with ciprofibrate therapy. However, the subsequent clinical course proved unfavorable.

Between 2019 and 2022, the patient experienced multiple hospitalizations for recurrent abdominal pain and acute pancreatitis, all occurring in the context of persistent severe hypertriglyceridemia. A portal venous phase contrast-enhanced abdominal CT scan performed subsequently demonstrated residual scar-like hypodense lesions in the pancreatic body, compatible with chronic sequelae of recurrent acute pancreatitis. Throughout this period, directly measured LDL and HDL cholesterol levels remained persistently low despite markedly elevated triglycerides, a pattern suggestive of an underlying chylomicronemia syndrome. Despite escalating combined treatment with statins, fibrates, and omega-3 fatty acids, triglyceride levels remained critically elevated, peaking at 4,350 mg/dL, demonstrating clear resistance to conventional lipid-lowering therapy (Table 1 and Figures 1-3).

**Table 1:** Serial lipid panel values. Follow-up periods are expressed relative to the index presentation (Month 0 = November 2019). Volanesorsen was initiated at Month 64 (March 2025). The table is divided into two sections: the pre-volanesorsen period (index presentation through Month 63) and the post-volanesorsen period (Month 64 onward). Clinical events are denoted by bracketed annotations next to the corresponding follow-up time point: (AP) = acute pancreatitis episode; (ABD) = abdominal pain without pancreatitis; (Vol ↓) = first measurement after volanesorsen initiation. A dash (-) indicates that the value was not available at that time point. TG: Triglycerides; Chol: Cholesterol; HDL: High-density lipoprotein; LDL: Low-density lipoprotein; HbA1c: Glycated hemoglobin; Creat.: Serum creatinine.

Follow-up period	TG (mg/dL)	Total chol. (mg/dL)	HDL (mg/dL)	LDL (mg/dL)	HbA1c (%)	Creat. (mg/dL)	Platelets (×10³/µL)
Pre-volanesorsen period (index presentation through Month 63)							
Baseline (AP)	1,294	-	-	-	5.46	0.96	363
Month 7	1,799	167	11	13	-	-	-
Month 9	1,448	154	7	14	-	-	-
Month 12	2,780	251	11	-	5.59	-	-
Month 18	3,957	-	-	-	5.38	1.04	-
Month 22	2,984	310	13	30	-	-	-
Month 24 (AP)	3,810	434	28	32	5.98	-	-
Month 29 (ABD)	2,800	303	16	-	-	0.88	246
Month 32	3,730	-	-	-	-	-	-
Month 41	4,350	-	-	-	-	-	-
Month 47	1,060	-	-	-	-	-	-
Month 50	2,340	197	9	12	-	-	-
Month 56	2,520	280	11	-	6.11	-	-
Month 57	2,850	282	11	34	-	-	-
Month 63	3,918	294	8	-	-	0.82	337
Post-volanesorsen period (Month 64 onward)							
Month 65 (Vol ↓)	915	76	5.1	111	-	0.93	192
Month 68	714	-	-	-	-	-	392
Month 71	885	-	-	-	-	-	179
Month 74	948	-	-	-	-	-	342

**Figure 1:**
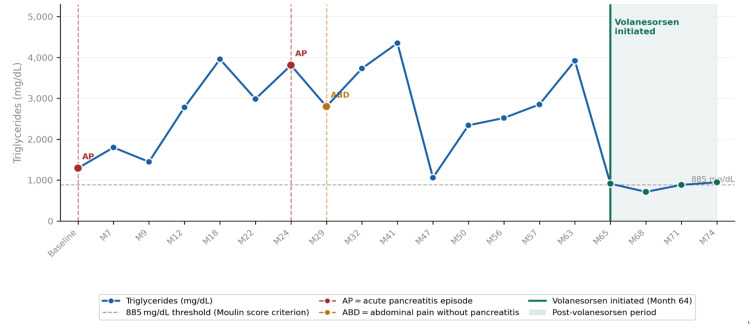
Longitudinal triglyceride profile. Serum triglyceride levels (mg/dL) over 74 months of follow-up. The dashed horizontal reference line indicates 885 mg/dL, corresponding to the Moulin score criterion. Colored points and vertical dashed lines denote clinical events. The shaded green area indicates the post-volanesorsen period. AP: Acute pancreatitis; ABD: Abdominal pain without pancreatitis. Month 0 = Index presentation; volanesorsen was initiated at Month 64. The figure was generated using Python (Matplotlib library). No AI-based image generation tools, infographic templates (Canva, BioRender, PowerPoint Designer), or similar platforms were used.

**Figure 2:**
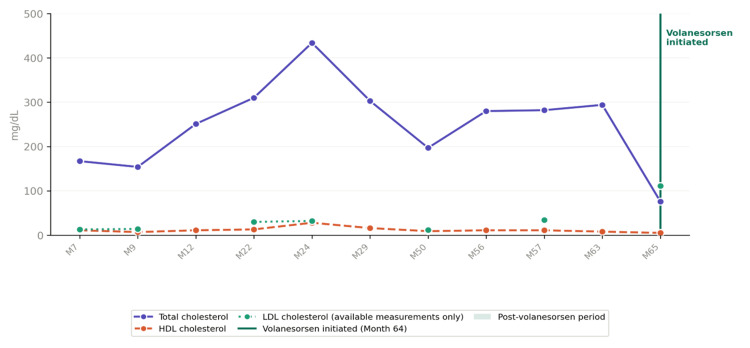
Longitudinal cholesterol profile. Total cholesterol, high-density lipoprotein (HDL), and low-density lipoprotein (LDL) cholesterol levels (mg/dL) over follow-up. LDL is plotted only at time points where the value was measurable (dotted line); segments between unavailable measurements are not interpolated. Note the persistently suppressed LDL and HDL levels during periods of severe hypertriglyceridemia, with LDL recovery to 111 mg/dL after volanesorsen initiation. The shaded green area indicates the post-volanesorsen period. Month 0 = index presentation; volanesorsen was initiated at Month 64. The figure was generated using Python (Matplotlib library). No AI-based image generation tools, infographic templates (Canva, BioRender, PowerPoint Designer), or similar platforms were used.

**Figure 3:**
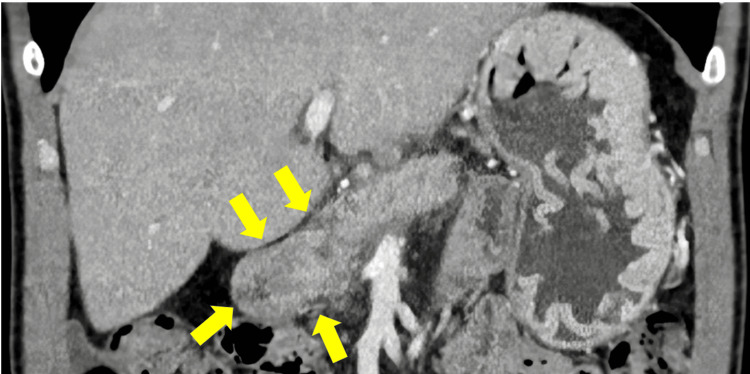
Contrast-enhanced abdominal CT, portal venous phase, coronal reconstruction. Yellow arrows indicate hypodense scar-like lesions within the pancreatic body and head, consistent with chronic sequelae of recurrent acute pancreatitis. The pancreatic parenchyma appears atrophic in the affected segments. No peripancreatic fluid collections or pseudocysts were identified.

Given the severity of the hypertriglyceridemia, recurrent pancreatitis, and inadequate therapeutic response, joint evaluation by the endocrinology and genetics departments was requested. Clinical scoring using the Moulin score yielded 10 points, classifying FCS as highly probable, based on fasting triglycerides exceeding 885 mg/dL on three consecutive measurements, at least one value ≥1,770 mg/dL, a history of pancreatitis, and recurrent abdominal pain.

Secondary causes of severe hypertriglyceridemia were systematically evaluated. The patient had no clinical diagnosis of diabetes mellitus; serial HbA1c measurements throughout follow-up ranged from 5.38% to 6.11% (Table 1), and urinalysis repeatedly showed no glucosuria or proteinuria. Alcohol consumption was denied at all clinical encounters. Thyroid function was assessed on multiple occasions, with thyroid-stimulating hormone (TSH) transiently elevated at 6.26 mIU/L in June 2019 and 4.82 mIU/L in November 2020, normalizing to 3.35 mIU/L by July 2024, consistent with subclinical hypothyroidism without overt dysfunction, for which no replacement therapy was initiated; severe hypertriglyceridemia persisted independently of TSH fluctuations across all time points. Renal function remained preserved throughout follow-up, with serum creatinine consistently below 1.1 mg/dL and absence of proteinuria on repeated urinalysis, excluding nephrotic syndrome. BMI was 25 kg/m², without clinical features of metabolic syndrome. HIV serology was non-reactive. Hepatic transaminases were consistently within normal or minimally elevated ranges. Notably, the patient had been on chronic valproic acid therapy for a psychiatric comorbidity, which was identified during the medication review as a potential aggravating factor for hypertriglyceridemia; however, given the extreme magnitude and refractoriness of the hypertriglyceridemia, this alone was considered insufficient to account for the observed phenotype, further supporting the clinical decision to pursue molecular genetic testing. Valproic acid was subsequently discontinued as part of a reassessment of the patient's psychiatric management.

In March 2024, next-generation sequencing (NGS) of a multigene panel targeting the five canonical genes associated with FCS (*LPL*, *APOC2*, *APOA5*, *LMF1*, and *GPIHBP1*), performed through Mendelics Genomic Analysis, identified a homozygous missense pathogenic variant in the *LPL* gene: NM_000237.3.644G>A, p.(Gly215Glu), also known by its legacy designation p.Gly188Glu, confirming the diagnosis of FCS.

Following diagnostic confirmation in March 2024 and failure of conventional therapies, treatment with volanesorsen was initiated in March 2025, a delay attributable to administrative and healthcare access barriers inherent to high-cost medication approval processes in Colombia. During this interval, triglyceride levels remained critically elevated, reaching 3,918 mg/dL in February 2025. The patient received volanesorsen as a 285 mg prefilled syringe administered subcutaneously every two weeks. After treatment initiation, a marked reduction in triglyceride levels was observed, from 3,918 mg/dL in February 2025 to 915 mg/dL by Month 65, with sustained levels below 950 mg/dL through Month 74 of follow-up (Table 1 and Figure 1). No new episodes of acute pancreatitis were documented during periods of uninterrupted volanesorsen therapy, platelet counts remained within normal limits throughout, and the patient reported significant improvement in quality of life. Written informed consent was obtained from the patient for the publication of this case report.

## Discussion

Familial chylomicronemia syndrome is an extremely rare autosomal recessive disorder caused by biallelic pathogenic variants in genes essential for chylomicron metabolism: *LPL* (encoding lipoprotein lipase), *APOC2* (encoding an essential *LPL* cofactor), *APOA5* (which facilitates chylomicron-LPL interaction), *LMF1* (required for proper LPL folding and secretion), and *GPIHBP1* (responsible for endothelial LPL transport and stabilization) [2]. Its estimated prevalence is approximately 1 per 300,000 individuals worldwide, though notably higher in founder populations such as Quebec, Canada, where it ranges from 1 in 10,000 to 1 in 30,000 [3,4].

In FCS, the functional absence of LPL prevents chylomicron catabolism, resulting in persistent fasting chylomicronemia, the characteristic lactescent plasma appearance, and extreme hypertriglyceridemia, often exceeding 1,500 mg/dL and frequently surpassing 10,000 mg/dL [5]. Reduced chylomicron remnant formation further limits hepatic very-low-density lipoprotein (VLDL) synthesis, leading to normal or low VLDL, LDL, and HDL concentrations [6], a pattern consistently observed in our patient, who maintained persistently low directly measured LDL and HDL levels throughout the entire follow-up period, despite markedly elevated triglycerides (Table 1, Figure 1, and Figure 2).

Clinically, FCS is characterized predominantly by recurrent abdominal pain and acute pancreatitis, a frequent and potentially life-threatening complication that may arise as early as childhood [7]. Pancreatitis is thought to result from the local release of toxic free fatty acids generated by intrapancreatic triglyceride hydrolysis, triggering inflammatory injury and tissue necrosis [7]. In our patient, recurrent pancreatitis with triglyceride levels persistently exceeding 4,000 mg/dL despite conventional therapy was the key clue prompting suspicion of FCS. The Moulin score, a validated clinical tool integrating biochemical and clinical variables to estimate FCS probability before genetic confirmation [8,9], yielded 10 points in our patient, classifying FCS as highly probable and supporting the decision to pursue molecular testing.

From a genetic standpoint, FCS is a heterogeneous disorder. *LPL* variants account for approximately 60-80% of cases and are associated with markedly reduced or absent enzymatic activity [5]. The variant NM_000237.3.644G>A, p.(Gly215Glu), also known by its legacy designation p.Gly188Glu, is classified as pathogenic in ClinVar (Variation ID: 1522), supported by in vitro and in vivo functional studies demonstrating a damaging effect on LPL enzymatic activity, and has been associated with FCS in both homozygous and compound heterozygous patients. Notably, a large British cohort of 880 individuals with severe hypertriglyceridemia identified this as the most frequent variant among *LPL*-FCS cases (41.7%), with a median of 17.5 years between symptom onset and genetic diagnosis and over 80% of patients experiencing acute pancreatitis, with 93% suffering recurrent episodes [10], a pattern mirrored in our patient and one that underscores the urgent need for early molecular testing. Orthogonal confirmation by Sanger sequencing and copy-number variant (CNV) analysis was not performed; accordingly, the possibility that the apparent homozygosity represents a hemizygous point variant combined with an undetected deletion on the second allele cannot be formally excluded.

The identification of a homozygous pathogenic *LPL* variant carries important implications for the patient’s family. Under the autosomal recessive inheritance pattern of FCS, both parents are obligate heterozygous carriers, each sibling has a 25% probability of being homozygous affected and a 50% probability of being a carrier, and the patient’s offspring are obligate carriers at minimum [2,3]. In this case, the patient reported 14 siblings, two of whom died prematurely from causes that were never clarified; given the clinical context, undiagnosed FCS cannot be excluded as a contributing factor in those deaths. Cascade genetic testing is therefore strongly recommended for all available first-degree relatives, including parents, siblings, and the patient’s two daughters, who have thus far declined screening [3,4]. This recommendation is further reinforced by the patient’s origin from a rural area with limited healthcare access, where affected relatives may remain undiagnosed and without access to preventive dietary intervention or clinical surveillance, circumstances that perpetuate the diagnostic delays and preventable disease burden that characterize FCS in underserved populations.

A screening study by Rodriguez FH et al. in Pereira, Colombia, conducted between 2010 and 2020 among adults with severe hypertriglyceridemia (≥500 mg/dL), found that only 2.4% met clinical criteria for FCS based on the Moulin score. Genetic analysis confirmed a single case, associated with APOA5 and GPIHBP1 variants, within a cohort of 2,415 patients, corresponding to an estimated prevalence of 0.41 per 1,000 individuals with triglycerides above 500 mg/dL [11]. More recently, Román-González A et al. published the first Colombian series of patients treated with volanesorsen, in which 70% carried LPL variants and experienced triglyceride reductions of 50-60%, with no further episodes of pancreatitis during follow-up [12]. Collectively, Colombian reports have documented variants in *LPL*, *APOC2* [13], *GPIHBP1* [14], and *APOA5* [11], reflecting the molecular heterogeneity of FCS in this population.

Management of FCS relies on strict dietary modification alongside alcohol avoidance and carbohydrate restriction to minimize chylomicron formation and reduce pancreatitis risk. Unlike multifactorial hypertriglyceridemia, conventional lipid-lowering therapies, such as fibrates and omega-3 fatty acids, show limited efficacy due to severely reduced or absent LPL function, as evidenced in our patient by persistent severe hypertriglyceridemia and recurrent pancreatitis despite prolonged conventional therapy. Of note, valproic acid, which the patient received chronically for a psychiatric comorbidity and was subsequently discontinued following recognition of its potential metabolic contribution, is a recognized secondary contributor to hypertriglyceridemia through inhibition of mitochondrial fatty acid β-oxidation, increased hepatic triglyceride synthesis via diacylglycerol O-acyltransferase 2 (DGAT2) upregulation, and valproate-induced hyperinsulinemia promoting lipogenesis [15]. However, valproate-induced hypertriglyceridemia typically manifests as mild-to-moderate elevation and is rarely sufficient to explain triglyceride levels of the magnitude observed in this patient, repeatedly exceeding 2,000-4,350 mg/dL, nor the characteristic suppression of LDL and HDL that defines the FCS lipid phenotype. This case therefore illustrates how a pharmacological secondary trigger may amplify an underlying monogenic lipid disorder and reinforces the importance of pursuing molecular testing even when a potential secondary cause is identified.

In this context, APOC3 inhibitors, including volanesorsen, olezarsen, and plozasiran, have demonstrated significant triglyceride reductions and a lower incidence of acute pancreatitis in patients with FCS [16,17]. In the phase 3 APPROACH trial, volanesorsen reduced fasting triglycerides by 77% at three months (mean absolute reduction of -1,712 mg/dL) compared to an 18% increase in the placebo group, with 77% of treated patients achieving triglyceride levels below 750 mg/dL versus 10% in the placebo arm [16]. Regarding pancreatitis, an exploratory adjudicated analysis over 52 weeks documented four episodes in three placebo patients compared to one episode in the volanesorsen group. Notably, thrombocytopenia was identified as a clinically relevant adverse effect, with platelet counts below 100,000/µL occurring in 45% of volanesorsen-treated patients versus none in the placebo group; accordingly, close platelet monitoring is mandatory during treatment. In our patient, platelet counts remained within normal limits throughout follow-up. Notably, valproic acid had already been discontinued prior to volanesorsen initiation, making its withdrawal an unlikely confounder for the biochemical response observed; the marked triglyceride reduction can therefore be more confidently attributed to volanesorsen itself. No new episodes of pancreatitis were documented during periods of uninterrupted therapy. Other therapeutic strategies, including lomitapide and ANGPTL3 inhibitors, remain under evaluation for selected patients [18,19].

This case also broadens the national experience with FCS and highlights the value of molecular diagnosis in patients with suggestive clinical features. Limitations include the absence of functional studies to directly assess residual LPL activity, the lack of Sanger confirmation and CNV analysis, which precludes formally excluding hemizygosity, and the absence of segregation data, all of which prevent definitive causal attribution of the phenotype to the identified variant alone. Additionally, the concurrent use of valproic acid throughout a significant portion of follow-up represents an inherent limitation in fully excluding a pharmacological contribution to the observed hypertriglyceridemia; however, the severity, pattern, and genetic confirmation of the phenotype support a primary monogenic etiology. Finally, the post-treatment follow-up period of approximately 10 months with sparse sampling points limits the characterization of the durability of the triglyceride response, and longer-term data will be needed to confirm its persistence.

## Conclusions

This case highlights that FCS should be actively suspected in patients with persistent severe hypertriglyceridemia and recurrent pancreatitis refractory to conventional therapy. Molecular diagnosis is essential to clarify the etiology, characterize the causative variant, and guide therapeutic decision-making. The favorable response to volanesorsen in this Latin American patient reinforces the efficacy of LPL-independent, precision medicine-based strategies and expands the molecular evidence on FCS in Colombia.
